# Homeopathic Medicines for the Treatment of Acute Otitis Media: a Real-World Cohort Study on Recurrences and Antibiotic Prescriptions Compared to those with Conventional Treatments

**DOI:** 10.1055/a-2727-3418

**Published:** 2026-03-12

**Authors:** Norbert Banik, Kristina Hammerstiel, Sabine Niederle, Thorsten Reineke

**Affiliations:** 1 Independent Consultant in Health Services Research and Epidemiology, München, Germany; 2 Department of Homeopathy, Deutsche Homöopathie-Union GmbH & Co. KG, Karlsruhe, Germany; 3 Department of Clinical Research, Dr. Willmar Schwabe GmbH & Co. KG, Karlsruhe, Germany

**Keywords:** acute otitis media, homeopathic medicines, conventional medicines, real-world data, cohort study

## Abstract

**Introduction:**

The study investigated the role of homeopathic medicines in daily treatment of uncomplicated (i.e., without need for an initial antibiotic prescription) acute otitis media (AOM). Recurrence rates and antibiotic prescriptions over 12 months were compared in patients initially prescribed either homeopathic medicines or medicines from one of three conventional therapeutic classes.

**Methods:**

This exploratory cohort study used real-world electronic health care data from the Disease Analyzer database (IQVIA). German patients of all ages diagnosed with AOM between 2010 and 2018 who were prescribed either homeopathic, nasal, otological medicines or non-opioid analgesics on the day of diagnosis or within 6 days thereafter were included. AOM recurrence was assessed using multivariable logistic regression; the number of antibiotic prescriptions was assessed using multivariable negative binomial regression.

**Key Results:**

A total of 113,983 of 745,372 patients diagnosed with AOM were eligible for analysis. Of these, 9.9% of patients initially received prescriptions for homeopathic medicines. In the total patient group, these prescriptions were associated with a slightly reduced risk of AOM recurrence compared with otological medicines (odds ratio, 1.17; statistically significant, *p* = 0.011). All results of the negative binomial regression analysis of antibiotic prescriptions were in favor of homeopathic medicines. Those were statistically significant in the comparison versus otological medicines in all patients and in the adult sub-group. Time-to-event analysis for first antibiotic prescriptions showed statistically significantly reduced time-related risks in the homeopathy group compared with all groups of conventional medicines included (in all patients and in two pre-defined age-dependent sub-groups).

**Conclusion:**

The real-world data analyzed in this study reveal that homeopathic medicines play a relevant role in daily AOM treatment as a stand-alone therapy class. Additionally, the study yielded important methodological findings on prescription patterns in routine AOM care in Germany, with specific focus on the role of homeopathic medicines.

## Introduction

Otitis media (OM), or middle ear inflammation, affects the middle ear cavity and ossicles. OM encompasses a wide range of conditions, such as acute otitis media (AOM) and OM with effusion (also known as “glue ear”). AOM usually develops as a sequela of an upper respiratory tract infection (URTI) which can be caused by different viruses and sometimes accompanied by a bacterial superinfection. 1 2 3

AOM is one of the most common causes for the use of medical services and the prescription of medication worldwide. A systematic review on the global impact of the disease estimates that the average incidence rate of AOM is 10.8 new episodes per 100 people annually. The actual number of cases varies considerably from region to region and correlates with those regions' relative prosperity. The highest global incidence rates of AOM are found in children under the age of 5. 3 4

Whilst the incidence of AOM has declined over the recent decades due to various measures such as the implementation of clinical guidelines advocating more stringent diagnostic criteria and, probably, the introduction of pneumococcal conjugate vaccines, 3 5 6 this disease is still common and is one of the leading causes of antibiotic prescriptions, especially in children. 7 8 9 10

Symptoms of AOM, such as inflammation, pain and fever, often subside spontaneously, again especially in children. Therefore, in many cases it seems advisable to consider antibiotic treatment only as part of a “watchful waiting” approach and treat the initial inflammatory symptoms first with another, more tolerable, medication. 1 It is recommended that AOM treatment is usually started with non-opioid analgesics, followed by a delayed prescription of antibiotics only in selected patients, if necessary. 2 Although there is no clinical recommendation in official guidelines for the use of topical or oral decongestants, antihistamines or corticosteroids, these are often used in AOM patients due to the distressing symptoms. 2 3 11

Apart from this, treatment with homeopathic medicines is another therapeutic option in cases of OM and e.g. URTI in all age groups. 12 13 Several studies with different designs and target parameters have been conducted to investigate the effectiveness of homeopathic medicines in the treatment of AOM. There have been some promising results, but due to some limitations and the heterogeneity of the studies, the evidence for the general benefits of homeopathic medicines in the treatment of OM is not yet sufficient. 12 14 15 16

There is also a lack of sufficiently large studies with real-world data. As a step to close this knowledge gap, we used real world prescription data in our study to investigate whether there is a difference in recurrence of AOM or antibiotic prescribing in patients initially treated with homeopathic medicines compared with those receiving conventional symptomatic therapy. The study also serves to investigate the usage patterns of homeopathic medicines in the routine treatment of AOM. The analysis is based on a large pharmacoepidemiological database from a patient cohort in Germany.

## Methods

### Database

The present exploratory study used data from the German Disease Analyzer database (IQVIA). This (not publicly accessible) database has already been extensively described in the literature. 17 To summarize, the Disease Analyzer database includes anonymized patient data on demographic variables, diagnoses and prescriptions obtained in general and specialized practices in Germany. Diagnoses are coded using the International Classification of Diseases, 10th revision (ICD-10). As the database does not contain any specific clinical information about the disease (e.g. degree of severity, symptoms present) beyond the current ICD-coded diagnosis at the time of prescription, no other detailed comparisons of patients and disease progression can be made. The quality of the data is assessed every month based on several criteria (completeness of information about sex and age, linkage between diagnoses and prescriptions). Practices contributing regularly to the database are selected according to the yearly statistics of the German Medical Association, which include information on the physician's age, specialty group, size of community, and German federal state. It has been shown in prior research that the Disease Analyzer database is representative of all practices in Germany. 17 In recent years, several epidemiological studies in the field of otorhinolaryngology using Disease Analyzer database have been published. 4 13 18 19 20

### Study Population

This study included patients with a first diagnosis of AOM, ICD-10 codes H65.0, H65.1, H65.9 (non-suppurative OM) or H66.0, H66.4, H66.9 (suppurative and non-specified OM), in the period between January 1, 2010 and December 31, 2018 (index date). This time frame was chosen to exclude the COVID-19 pandemic period, which significantly changed the general infection situation and routine medical care. 21 22 23

On the day of diagnosis and for up to 6 days thereafter, the patients included in the study had received prescriptions exclusively for medicines from one of four therapeutic classes: homeopathic medicines, conventional nasal (topical/systemic) medicines, otological medicines or non-opioid analgesics. The prescribing physicians selected the treatment freely on an individual basis according to their judgment. Retrospectively only, for the sake of this study, the individual treatments have been grouped according to the following four therapeutic classes: (1) homeopathic medicines (single or combination medicines); (2) nasal medicines (topical/systemic) including chemically based decongestants and corticosteroids for topical use, as well as sympathomimetics for topical or systemic use; (3) otological medicines including chemically based anti-infectives, corticosteroids, as well as analgesics and anesthetics for local use; and (4) non-opioid analgesics including NSAIDs relevant in AOM therapy ( Supplementary Table S1 , available in the online version only). These initial prescriptions determined group membership for further evaluation in our study. During the one-year follow-up period, there were no restrictions on follow-up prescriptions and those, if applicable, are not recorded for this study.

The medicines included in the study were classified according to WHO Anatomical-Therapeutical-Chemical classes (shown in Supplementary Table S1 , available in the online version only). Only patients with an “uncomplicated” AOM, in the sense that they initially did not need an antibiotic, were included. This definition is consistently applied in the text whenever the term “AOM” is used in relation to the study population. Follow-up data were collected for a period of 365 days after the index date as far as data were available for the respective patient.

The following patient exclusion criteria were applied:

- prescriptions of medicines for AOM complaints from more than one therapeutic class at index date,
- AOM diagnosis within the 90 days prior to the index date,
- prescriptions of medicines from at least one of the study's therapeutic classes within 90 days prior to the index date,
- prescription of an antibiotic in the 90 days before to 13 days after the index date,
- prescriptions of anti-neoplastic or immunomodulatory medicines in the time from 90 days before to 365 days after the index date,
- unknown sex or age,
- patients whose medical practice did not deliver data continuously for a given patient for a least 3 months before to 12 months after the index date.

### Study Outcome

The study compared the association of the prescribed therapy class in AOM with:

- the risk of first AOM recurrence (new diagnosis after index date),
- time to first AOM recurrence,
- risk of, and number of, antibiotic prescriptions during follow-up,
- time to first antibiotic prescription.

These outcomes were assessed as relative effects of each of the three investigated groups receiving conventional medicines versus the group receiving homeopathic medicines. Each patient was followed up for up to 365 days after the index date. The analysis period for the outcomes was 14–365 days after the index date.

### Statistical Analyses

As the group of patients treated with homeopathic medicines is compared with defined conventional medicine classes, the homeopathy group is set as reference group in all analyses. The three conventional groups are referred to hereafter as “nasal,” “otological” and “non-opioid analgesics” groups, respectively.

Multivariable logistic regression was used to analyze the association between therapy class and first AOM recurrence. The data were adjusted for age groups, sex, health insurance coverage, season of index date (quarterly intervals) and specialty of diagnosing/prescribing doctor (general practitioner (GP), ear, nose & throat (ENT) specialist, pediatrician). The odds ratios (ORs) and their 95% confidence intervals (95% CIs) were calculated between the reference and the analysis groups. Due to the exploratory character of the study, descriptive *p* -values are also reported.

A random effect for practice identifier was included in the model to control for possible associations between the likelihood of individual doctors having a greater or lesser inclination to choose homeopathic medicines in treating the AOM and their likelihood to also diagnose recurrence. Since the patients were observed for a standardized period after index date, there was no need to adjust the results for differential patient follow up.

The analysis of the mean number of antibiotic prescriptions during follow-up (i.e., between day 14 and day 365 after the index date) was generally similar to that of the recurrence of the first AOM in terms of the variables used for adjustment.

The main difference between the analyses of our two outcome measures (recurrence of any AOM and number of antibiotic prescriptions) was the structure of the outcome variable. Whilst the first analysis addresses a binary variable (recurrence of any AOM), the second analysis aims at the number of antibiotic prescriptions, which are rare events. Usually, a Poisson distribution is used as a model for rare events, and consequently a Poisson regression instead of a logistic regression would have been more applicable. However, since the Poisson distribution assumes that the mean is equal to the variance (one-parameter distribution) and this assumption may have been violated, a negative binomial model was used instead. Again, a random effect for the practice identifier was included in the model and no adjustment for different observation time was required.

The time to occurrence of the first AOM diagnosis after index date (“AOM recurrence”) and time to first antibiotic prescription during follow-up was further specified by multivariable Cox regression analyses, adjusted as described above. Results of the Cox regression models are given as the hazard ratio (HR) between the reference and the analysis groups.

A nominal *p* -value of <0.05 is considered statistically significant (in an exploratory sense); no adjustment for multiplicity was made. All analyses were carried out using SAS Version 9.4 and were based on a pre-established study protocol.

## Results

### Baseline characteristics

**Fig. 1:**
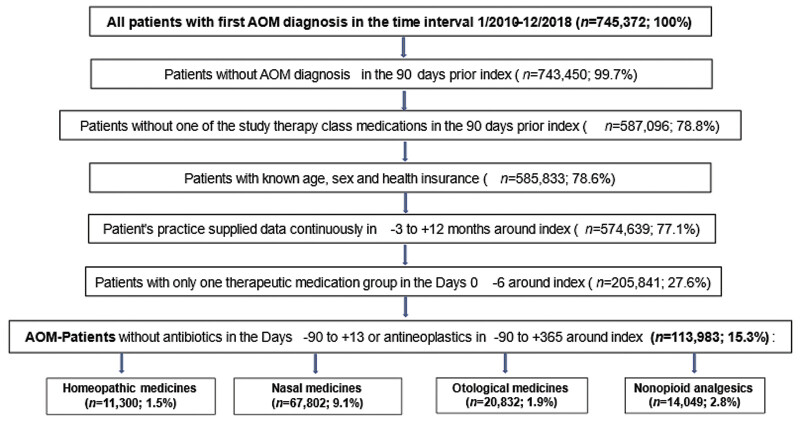
Selection of overall study patients for comparison of the therapeutic medication groups according to inclusion and exclusion criteria. Respective percentage values refer to the initial AOM cohort. AOM, acute otitis media.

Initially, a total of 745,372 patients diagnosed with AOM between January 1, 2010 and December 31, 2018 were identified in the database. 113,983 patients (15.3%) were eligible for analysis ( Fig. 1 ). Of the study population analyzed, 9.9% of patients received prescriptions for homeopathic medicines. In comparison, nasal medicines were prescribed to 59.5%, otological medicines to 18.3% and non-opioid analgesics to 12.3% of patients ( Table 1 ). Among the prescribed homeopathic medicines, both single remedies and combination preparations were included. Some combination preparations have approved indications. The numbers of children and adolescents (0–17 years, *n* = 59,058, 51.8%) and adults (18–75+ years, *n* = 54,925, 48.2%) in the overall study group were comparable. Analysis of the age distribution in each of the various therapy groups revealed clear differences from this general distribution in all groups except in the nasal medicine group. There was an inverse relationship between the proportion of children and adolescents as compared with the proportion of adult patients in the homeopathy group and the otological group. Non-opioid analgesics, which are also frequently used to treat fever, were predominantly prescribed (75.0%) to children and adolescents ( Table 1 ).

**Table 1:** Baseline characteristics of included study patients with acute otitis media diagnosis

Therapy class	Homeopathic medicines	Nasal medicines (topical/systemic)	Otological medicines	Non-opioid analgesics	Overall patients counts
	*N* (%) a	*N* (%) a	*N* (%) a	*N* (%) a	*N* (%)
Total patient counts	11,300 (9.9)	67,802 (59.5)	20,832 (18.3)	14,049 (12.3)	113,983 (100.0)
Index age class					
0–5 years	5,360 (47.4)	21,138 (31.2)	2,730 (13.1)	6,587 (46.9)	35.815 (31.4)
6–11 years	1,747 (15.5)	8,791 (13.0)	2,076 (10.0)	2,852 (20.3)	15,466 (13.6)
12–17 years	595 (5.3)	4,457 (6.6)	1,632 (7.8)	1,093 (7.8)	7,777 (6.8)
* 0–17 years*	*7,702 (68.2)*	*34,386 (50.7)*	*6,438 (30.9)*	*10,532 (75.0)*	*59,058 (51.8)*
18–24 years	408 (3.6)	3,951 (5.8)	1,518 (7.3)	679 (4.8)	6,556 (5.8)
25–49 years	1,660 (14.7)	14,379 (21.2)	5,753 (27.6)	1,833 (13.0)	23,625 (20.3)
50–74 years	1,267 (11.2)	12,101 (17.8)	5,371 (25.8)	829 (5.9)	19,568 (17.2)
≥75 years	263 (2.3)	2,985 (4.4)	1,752 (8.4)	176 (1.3)	5,176 (4.5)
* 18–75+ years*	*3,598 (31.8)*	*33,416 (49.3)*	*14,394 (69.1)*	*3,517 (25.0)*	*54,925 (48.2)*
Patient sex					
Male	5,192 (45.9)	32,495 (47.9)	10,426 (50.0)	6,831 (48.6)	54,944 (48.2)
Female	6,108 (54.1)	35,307 (52.1)	10,406 (50.0)	7,218 (51.4)	59,039 (51.8)
Patient health insurance					
Statutory	9,422 (83.4)	59,471 (87.7)	18,948 (91.0)	12,733 (90.6)	100,574 (88.2)
Private	1,878 (16.6)	8,331 (12.3)	1,884 (9.0)	1,316 (9.4)	13,409 (11.8)
Season of index date					
Months 12 to 2	3,644 (32.2)	21,435 (31.6)	5,231 (25.1)	4,726 (33.6)	35,036 (30.7)
Months 3 to 5	3,396 (30.1)	19,500 (28.8)	4,790 (23.0)	3,649 (26.0)	31,335 (27.5)
Months 6 to 8	1,875 (16.6)	11,017 (16.2)	5,594 (26.9)	2,397 (17.1)	20,883 (18.3)
Months 9 to 11	2,385 (21.1)	15,850 (23.4)	5,217 (25.0)	3,277 (23.3)	26,729 (23.5)
Doctor specialty					
GP	1,823 (16.1)	6,067 (8.9)	8,323 (40.0)	3,682 (26.2)	19,895 (17.5)
Pediatrician	2,520 (22.3)	16,787 (24.8)	1,607 (7.7)	7,567 (53.9)	28,481 (25.0)
ENT specialist	6,957 (61.6)	44,948 (66.3)	10,902 (52.3)	2,800 (19.9)	65,607 (57.6)

a In the first data line, percentage is in relation to total population over all therapy classes. In all other lines of the ‘medicine columns’, percentages are in relation to the total population within a therapy class.

There were similar numbers of male and female patients in the total study population, and this roughly applies for the sub-groups. The proportion of privately insured patients was highest in the homeopathic group (16.6%; compared with 12.3% in the nasal, 9.4% in the non-opioid analgesics, and 9.0% in the otological groups). For all groups except the otological group, the index dates fluctuated during the quarterly intervals, with the highest proportion of index dates observed in the winter months December–February (range: 31.6–33.6%). The otological group showed a more balanced distribution throughout the whole year (range: 23.0–26.9%), with the highest proportion in the summer months of June-August. The group of children and adolescents accounted for 51.8% of patients, but only 25.0% of this age group was treated by pediatricians. This is even less than the total percentage of children under the age of 6 (31.4%), who would be most likely to be treated by a pediatrician. More than half (57.6%) of all patients were treated by ENT specialists. They had the largest proportion of patients in all medicine groups except for non-opioid analgesics, where pediatricians dominated ( Table 1 ). All specialist groups equally prescribed homeopathic medicines for approximately 10% of their respective patients. In all other therapy groups, there were some major differences in the specialist group prescriptions (data not shown).

The inclusion criterion, that initially only medicines from a single therapeutic group were allowed, had the greatest impact on the number of patients otherwise eligible for analysis, as it reduced numbers by approximately 50% ( Fig. 1 ). This has implications for the generalizability of our study to a total AOM population, which will be discussed later. However, this restriction to monotherapy classes is a methodological requirement to be able properly to address the aim of the study.

### Acute Otitis Media Recurrence

At the end of the follow-up period (12 months after index date), the incidence rate of patients with at least one AOM recurrence ranged from 12.1 to 18.0% ( Table 2 ). Results of multivariable logistic regression analyses, adjusted for any potential effects of the specified covariates, showed that homeopathic medicine prescriptions were associated with a significantly lower risk of AOM recurrence as compared with otological medicines in the total population (OR, 1.17; 95% CI, 1.04 to 1.33; *p* = 0.011) as well as in adult patients (OR, 1.56; 95% CI, 1.19 to 2.04; *p* = 0.001). In contrast, the otological group had a significantly lower risk in children and adolescents when compared with the group treated with homeopathic medicines (OR, 0.86; 95% CI, 0.75 to 0.99; *p* = 0.036). All other medicine groups showed comparable, non-significant results in all age groups ( Table 2 ).

**Table 2:** Results of the multivariable logistic regression of recurrence of acute otitis media between days 14 and 365 after index date

	All patients			Children and adolescents			Adult patients		
Treatment	Patients with event (%)	OR (95% CI)	*p* -Value	Patients with event (%)	OR (95% CI)	*p* -Value	Patients with event (%)	OR (95% CI)	*p* -Value
Homeopathic medicines	18.0	Reference	NA	23.1	Reference	NA	7.0	Reference	NA
Nasal medicines	15.3	1.05 (0.93-1.19)	0.398	23.0	1.09 (0.95-1.24)	0.224	7.4	1.01 (0.78-1.33)	0.920
Otological medicines	12.1	1.17 (1.04-1.33)	0.011	16.1	0.86 (0.75-0.99)	0.036	10.3	1.56 (1.19-2.04)	0.001
Non-opioid analgesics	16.5	0.97 (0.84-1.11)	0.627	19.9	0.96 (0.83-1.12)	0.643	6.4	1.07 (0.79-1.46)	0.656

Abbreviations: AOM, acute otitis media; CI, confidence interval; NA, not applicable; OR, odds ratio.

Note: Results were adjusted for age groups, sex, patient health insurance coverage, season of index date and specialty of diagnosing/prescribing doctor.

The multivariate time-to-event analyses revealed, as expected, results very much in line with the aforementioned multivariate results of the frequency of AOM recurrence ( Table 3 ). Both the relative frequencies and the timing of recurrences support a true effect in the same direction. If the fewer recurrences in the homeopathic medicines group had occurred earlier on average, this would have questioned the relevance of the frequency findings. However, this is not the case.

**Table 3:** Results of the multivariable Cox regression of time to first recurrence of acute otitis media between days 14 and 365 after index date

	All patients		Children and adolescents		Adult patients	
Treatment	HR (95% CI)	*p* -Value	HR (95% CI)	*p* -Value	HR (95% CI)	*p* -Value
Homeopathic medicines	Reference	NA	Reference	NA	Reference	NA
Nasal medicines	1.05 (1.00–1.10)	0.051	1.08 (1.02–1.13)	0.006	1.01 (0.89–1.15)	0.888
Otological medicines	1.13 (1.06–1.20)	<0.001	0.86 (0.80–0.93)	<0.001	1.52 (1.33–1.74)	<0.001
Non-opioid analgesics	0.96 (0.90–1.02)	0.203	0.96 (0.90–1.03)	0.223	1.06 (0.89–1.28)	0.507

AOM, acute otitis media; CI, confidence interval, HR, hazard ratio; NA, not applicable.

Results were adjusted for age groups, sex, patient health insurance coverage, season of index date and specialty of diagnosing/prescribing doctor.

### Antibiotic Prescriptions

The overall number of antibiotic prescriptions during follow-up was low in all groups: only 11.6 to 18.6% of patients received an antibiotic prescription ( Table 4 ). A total of 8.4 to 12.8% of patients received one, and 2.3 to 3.9% of patients received two antibiotic prescriptions (data not shown).

**Table 4:** Results of the multivariable Cox regression of time to 1 ^st^ antibiotic prescription between days 14 and 365 after index date

	All patients			Children and adolescents			Adult patients		
Treatment	with AB prescription (%)	HR (95% CI)	*p* -Value	with AB prescription (%)	HR (95% CI)	*p* -Value	with AB prescription (%)	HR (95% CI)	*p* -Value
Homeopathic medicines	11.6	Reference	NA	13.5	Reference	NA	7.6	Reference	NA
Nasal medicines	12.1	1.21 (1.14–1.28)	<0.001	16.6	1.21 (1.13–1.29)	<0.001	7.5	1.25 (1.10–1.42)	0.001
Otological medicines	15.7	1.43 (1.33–1.53)	<0.001	14.5	1.19 (1.09–1.30)	<0.001	16.2	1.63 (1.43–1.85)	<0.001
Non-opioid analgesics	18.6	1.17 (1.09–1.25)	<0.001	18.9	1.13 (1.05–1.23)	0.001	17.4	1.36 (1.18–1.57)	<0.001

Abbreviations: AB, antibiotic; CI, confidence interval; HR, hazard ratio; NA, not applicable.

Note: Results were adjusted for age groups, sex, patient health insurance coverage, season of index date and specialty of diagnosing/prescribing doctor.

Multivariate negative binomial regression analyses showed lower mean numbers of antibiotic prescriptions in patients in the homeopathy group (total population as well as children and adolescents) compared with patients in all other medicine groups. For all patients, the results were statistically significant in favor of the homeopathy group compared with the otological group (means ratio, 1.46; 95% CI, 1.20 to 1.77; *p* < 0.001) as well as for the sub-group of adult patients (means ratio, 1.68; 95% CI, 1.26 to 2.25; *p* = 0.001). In the sub-group of children and adolescents the same trend was found, not reaching statistical significance. Compared with the non-opioid analgesics group, the mean number of antibiotic prescriptions was significantly lower in the homeopathy group for the adult patients (means ratio, 1.46; 95% CI, 1.07 to 1.97; *p* = 0.016; Table 5 ).

**Table 5:** Results of multivariable negative binomial regression of the number of antibiotic prescriptions between days 14 and 365 after index date

	All patients				Children and adolescents				Adult patients			
Treatment	Total number of AB pre-scriptions	Mean number of AB pre-scription per patient (SD)	Means ratio (95% CI)	*p* -Value	Total number of AB pre-scriptions	Mean number of AB pre-scription per patient (SD)	Means ratio (95% CI)	*p* -Value	Total number of AB pre-scriptions	Mean number of AB pre-scription per patient (SD)	Means ratio (95% CI)	*p* -Value
Homeopathic medicines	1,860	0.16 (0.53)	Reference	NA	1,484	0.19 (0.57)	Reference	NA	376	0.10 (0.42)	Reference	NA
Nasal medicines	11,650	0.17 (0.55)	1.21 (1.0–1.47)	0.053	8,332	0.24 (0.66)	1.20 (0.98–1.48)	0.077	3,316	0.10 (0.40)	1.26 (0.95–1.68)	0.109
Otological medicines	4,765	0.23 (0.64)	1.46 (1.20–1.77)	<0.001	1,324	0.21 (0.62)	1.20 (0.98–1.49)	0.082	3,438	0.24 (0.65)	1.68 (1.26–2.25)	0.001
Non-opioid analgesics	3,845	0.27 (0.70)	1.19 (0.99–1.42)	0.067	2,942	0.28 (0.69)	1.13 (0.93–1.37)	0.226	902	0.26 (0.73)	1.46 (1.07–1.97)	0.016

Abbreviations: AB, antibiotic; CI, confidence interval; NA, not applicable; SD, standard deviation.

Notes: Results of the means ratios are adjusted for age groups, sex, patient health insurance coverage, season of index date and specialty of diagnosing/prescribing doctor. The *p* -values refer to the corresponding estimated means ratios.

Table 4 additionally shows the results of multivariable Cox regression analysis for the time to first antibiotic prescription. In the total population and in all sub-groups homeopathic medicines were associated with lower time-related risk of antibiotic prescriptions when compared with nasal and otological medicines as well as non-opioid analgesics. All results are descriptively statistically significant ( *p* = 0.001 or <0.001). Thus, for any antibiotic prescription that was made during follow-up, it came in general later for the homeopathic medicines group as compared with any conventional medicines.

## Discussion

The results of the present real-world cohort study with nearly 114,000 patients indicate that the use of homeopathic medicines plays a relevant role in the management of uncomplicated AOM in Germany. A total of 9.9% of patients received prescriptions for homeopathic medicines as an initial stand-alone treatment class, largely prescribed by ENT specialists. The study further showed that homeopathic and conventional medicines yield comparable outcomes regarding recurrence or subsequent antibiotic prescriptions.

The differences in the overall percentage of patients with recurrence are rather small for all comparisons but more pronounced for the homeopathy group compared with the otological group, where they reached statistical significance. These significant results are in favor of the homeopathic medicines for the total patient population as well as for the adult sub-group, whereas for the sub-group of children and adolescents it reveals the opposite direction ( Table 2 ). The latter can be explained mainly by the different relative distribution of children and adolescents compared with adults in the respective therapy groups (relative frequency of very young children in the homeopathic group is about 3-fold compared with the otological group). Since children, especially young children, suffer from more frequent AOM infections, this fact seems to have a therapy-independent influence on the overall results despite the adjustments made in the multivariable models. Time-related risk of first recurrence of AOM within 1 year after initial AOM diagnosis is similar to the results about the relative risks in all studied therapeutic classes, thus supporting the above findings.

Analysis of the frequency and time-related risks for first antibiotic prescription during follow-up revealed a slight but significantly lower risk for patients in the homeopathy group compared with all other medicine groups. Regarding the frequency of antibiotic prescriptions, there were some significant differences between the treatment groups in favor of homeopathy. In addition, antibiotics were prescribed significantly later in the homeopathy group than in the conventional therapy groups, both in the overall patient group and in both age sub-groups.

Our overall result is supported by the recently published results of an open-labeled, randomized controlled clinical trial comparing homeopathic and conventional treatment of AOM in 222 children, among others, in terms of recurrence and antibiotic prescribing during individual 12-month follow-up. 24 In that Indian trial, a statistically significant reduction in combined AOM symptom scores was observed in patients individually treated with homeopathic medicines ( *n* = 116) compared with the conventionally treated patients ( *n* = 106) on day 3 and day 7 of treatment. There was no statistical difference between the treatment groups on days 10 and 21. That trial revealed no higher risk of complications for homeopathically treated patients compared with conventionally treated patients. Such an evaluation was not possible with our retrospective study design based on real-world data.

A treatment model was developed for the Indian trial that enabled a qualified ENT-based diagnosis and treatment as well as individualized homeopathic treatment in an outpatient setting. However, the authors also point out the fundamental difficulties of studies on the treatment of acute indications such as AOM in an outpatient setting. This applies in particular to studies with children, which require a large time commitment from the parents.

A recent overview described the overall evidence from studies evaluating the use of homeopathic medicines in the treatment of AOM as not yet sufficient, due to study heterogeneity. 15 This is also addressed by Nathan et al., who suggest the use of homeopathic medicines, possibly in combination with conventional medicine, in the treatment of the acute symptoms of AOM during the watchful waiting period. 25 It might be an option also for other acute infections. A recent study published by Oberbaum et al. has shown that homeopathic therapy, supported by conventional medicine as a safety backup, is a useful, safe and cost-effective method for treating acute illnesses such as respiratory diseases and diarrhea in the primary care of infants up to the age of 2 years. 26 According to the authors, homeopathic treatment was more effective than conventional treatment in preventing sick days, episodes of illness and respiratory disease in the first 24 months of life.

In certain acute illnesses, such as URTIs of viral origin, antibiotic therapy is not indicated yet frequently prescribed in practice. 10 In such cases treatment with homeopathic medicines may be used effectively as monotherapy. Concomitant treatment with homeopathic medicines can reduce the severity of the symptoms, significantly shorten the duration of the illness and save on chemical–synthetic medicines. Based on the available literature, adverse drug reactions to homeopathic medicines are rare compared with conventional medicines and are usually mild to moderate and temporary. 14 27 28 29 30 This suggests that the integration of homeopathic medicines into primary care can be a valuable addition to conventional care, especially for vulnerable patients such as young children. 26 31

As mentioned above, the incidence rate of AOM is highest in children under 5 years of age. 3 4 5 This is reflected in our study, in which this age group accounts for 31.4% of patients. Nevertheless, the indication is also relevant in adult patients, which account for 48.2% of the total study population. A recent four-year Dutch study on AOM incidences in (adult) patients aged 15 years and older reported that the incidence continues to decrease with age (from 7.1/1,000 person-years in patients aged 15–39 years to 2.7/1,000 person-years in patients aged 64 years and older). 32 This is also reflected in our data ( Table 1 ). In the Dutch study, oral antibiotics were prescribed in 46.0% of all episodes and topical antibiotics in 21.2%. In our study, no antibiotics were allowed to be used at the start of treatment. During the one-year follow-up period, the number of patients prescribed antibiotics was between 11.6% and 18.6% in the respective treatment groups.

In the present study, all three specialist groups prescribed homeopathic medicines to approximately 10% of their respective patients. In an analogously conducted study of more than 610,000 patients suffering from various acute URTIs, it was found that the prescription rates for general practitioners and ENT specialists were also approximately 10% each. 13 Both studies also indicate that a relevant proportion of ENT specialists use these medicines in patients with uncomplicated AOM as well as in patients with acute URTI. The prescription rates are particularly noteworthy as these two studies only included patients who did not receive multiple prescriptions, but only medication from one therapeutic group at the start of the treatment.

In young children in particular, it is recommended to treat pain and fever with non-opioid analgesics to relieve symptoms quickly. 2 3 This is reflected in the prescription figures in our study. A total of 75.0% of analgesics were prescribed for children and adolescents, and pediatricians dominated this treatment group with 53.9%. The use of decongestant nasal drops in AOM is often viewed critically but can be helpful due to the frequent concomitant rhinosinusitis. 2 At 59.5%, they accounted for most prescriptions, and their use was comparable in frequency in the two respective age sub-groups: children/adolescents and adults ( Table 1 ).

### Strengths and Limitations

The current study was conducted comparably to the recently published acute URTI study. 13 Both studies applied a generally identical methodological approach; thus, the strengths and limitations mutually correspond. These studies differ only in the index diagnosis, and in the prescriptions of medicines for the indication being investigated.

One of the strengths of the study is the utilization of routine data from medical practices where diagnoses and prescriptions are continuously documented. The study is based on the Disease Analyzer database whose reliability and general representativeness have been proven in several studies, which supports the validity of our findings. It uses the documented ICD-10 code for the selection of the patient population, as the database itself does not contain detailed clinical information. The advantages (as well as the disadvantages) of the use of ICD-10 codes are discussed, using the example of inner ear diseases, by Seidel et al. 4 Another strength of this study lies in the large patient sample (∼114,000 patients), which includes children and adolescents, as well as adults, and in the inclusion of three different specialist groups (general practitioners, ENT specialists, pediatricians). The data show the prescription of homeopathic medicines in AOM treatment and clearly demonstrate the therapeutic relevance of these medicines, especially in ENT practices, and in children under 5 years of age. Our real-world data study is therefore a step toward closing the information gap regarding the use of homeopathic medicines compared with conventional treatment of uncomplicated AOM in everyday clinical practice in Germany.

Retrospective analyses of primary care databases are methodologically limited in terms of the accuracy and completeness of data. A range of limitations illustrates the additional difficulties in studies with non-prescription medicines using secondary data. One results from the fact that most of the primary prescribed medicines analyzed are, in principle, over-the-counter medicines. Nevertheless, a physician can issue a prescription if necessary and these prescriptions are documented. Consequently, in our study, information on medicines used without a prescription is missing in the database. Even if a medicine is prescribed by a physician in this given setting, there is no confirmation that patients actually take it and in what quantity. Also, the database does not contain information on the severity grade of AOM, nor information on typical clinical outcome parameters such as symptom duration and severity during follow-up or on quality of life. Therefore, the chosen criteria, AOM recurrence and prescription of antibiotics for AOM during follow-up, are a bridging approach to assess treatment outcomes in the given setting.

Data on socioeconomic status and lifestyle-related risk factors (smoking, alcohol, physical activity) are also not available, and the possibility of residual confounding by these and other factors cannot be excluded. Moreover, the observation of patients is limited to a single practice each. Therefore, any diagnoses or prescriptions patients may have received from other doctors are not recorded. Since most patients probably use medicines from more than one therapeutic class to treat their AOM in daily practice, the analyzed dataset cannot be considered representative for the entirety of patients with AOM in Germany. However, the allocation of patients into single therapeutic classes (i.e. excluding combinations of different classes) had methodological reasons. The design was chosen to facilitate the interpretation of the results in relation to the different therapeutic pathways.

## Conclusion

This study of nearly 114,000 patients provides comparative, real-world evidence on the use of homeopathic and conventional medicines in the treatment of children and adolescents as well as adults with uncomplicated AOM. It thus provides an insight into the relevance of homeopathic treatment for everyday practice in a common acute illness that is still frequently treated with inappropriate antibiotic therapy. Despite some inherent methodological limitations of the database used, the results show that homeopathic medicines are relevant for treatment and suggest that they are a valuable therapeutic option for this condition. Their good tolerability described in the literature and their potential contribution to reducing unnecessary antibiotic prescriptions in patients with AOM should also be considered within the wider context of health care decision-making. This is particularly important in view of the large number of affected children aged up to 5 years. Further research utilizing different study designs and databases is needed to expand the real-world evidence for homeopathy.

## Highlights

- Prescription data of nearly 140,000 German patients were used retrospectively to compare the use of homeopathic medicines with conventional symptomatic therapy in patients with acute otitis media.
- Similar overall results concerning recurrence and antibiotic prescriptions during individual 12-month follow-up were found for the patients with an initial treatment with homeopathic medicines compared with those treated with conventional therapy.
- The statistically significant results shown indicate a favorable outcome for homeopathic medicines.
- The data indicate that homeopathic medicines as stand-alone therapy, alongside conventional medicines, play a relevant therapeutic role in treating AOM in Germany.
