# Supplementary material for: Homeopathic Medicines for the Treatment of Acute Otitis Media: a Real-World Cohort Study on Recurrences and Antibiotic Prescriptions Compared to those with Conventional Treatments
Source: Homeopathy. 2026 Mar 12;115(3):135–43. doi: 10.1055/a-2727-3418 (PMC13412704; doi:10.1055/a-2727-3418)
Supplement: Supplementary file 1 — Supplementary Material [file 10-1055-a-2727-3418_29259146.pdf]

Supplementary Table S1. Assignment of ATC codes to medicine-classes relevant for the analyses (all ATC codes given as WHO ATC codes, adapted to the official German version 2022)

Group	ATC classes
**Homeopathic medicines**<br>*(single or combination medicines)*	V60A, N02BH, R01AH, R01BH, S02DH
**Nasal medicines** (topical/systemic)<br>*(including chemically based decongestants and corticosteroids for topical use, as well as sympathomimetics for topical or systemic use)*	R01 excluding R01AH, R01BH (both homeopathic medicines), R01AC (antiallergens), R01AP, and R01BP (both phytotherapeutic medicines)
**Otological medicines**<br>*(including chemically based antiinfectives, corticosteroids, as well as analgesics and anesthetics for local use)*	S02A-S02C, S02DA
**Nonopioid analgesics**<br>*(incl. NSAIDs relevant in AOM therapy)*	M01AE, N02B excluding N02BH (homeopathic medicines), and N02BP (phytotherapeutic medicines)

AOM: Acute Otitis Media; ATC: Anatomical Therapeutic Chemical; NSAIDs: Nonsteroidal Anti-Inflammatory Drug(s); WHO: World Health Organization.

To identify antibiotics, the ATC-code J01 was used.
